# Rational Pharmacological Approaches for Cognitive Dysfunction and Depression in Parkinson’s Disease

**DOI:** 10.3389/fneur.2015.00071

**Published:** 2015-03-31

**Authors:** Maritza Sandoval-Rincón, Michel Sáenz-Farret, Adán Miguel-Puga, Federico Micheli, Oscar Arias-Carrión

**Affiliations:** ^1^Unidad de Trastornos del Movimiento y Sueño, Hospital General Ajusco Medio, Secretaría de Salud, Mexico City, Mexico; ^2^Programa de Parkinson y Movimientos Anormales, Hospital de Clínicas “José de San Martín,” Universidad de Buenos Aires, Buenos Aires, Argentina; ^3^Unidad de Trastornos del Movimiento y Sueño, Hospital General Dr. Manuel Gea González, Secretaría de Salud, Mexico City, Mexico

**Keywords:** Parkinson’s disease, cognitive disorders, depression, major depressive disorder, dementia, drug therapy, non-motor symptoms

## Abstract

Parkinson’s disease (PD) is not a single entity but rather a heterogeneous neurodegenerative disorder. The present study aims to conduct a critical systematic review of the literature to describe the main pharmacological strategies to treat cognitive dysfunction and major depressive disorder in PD patients. We performed a search of articles cited in PubMed from 2004 to 2014 using the following MeSH terms (Medical subject headings) “Parkinson disease”; “Delirium,” “Dementia,” “Amnestic,” “Cognitive disorders,” and “Parkinson disease”; “depression,” “major depressive disorder,” “drug therapy.” We found a total of 71 studies related to pharmacological treatment in cognitive dysfunction and 279 studies for pharmacological treatment in major depressive disorder. After fulfillment of all the inclusion and exclusion criteria, 13 articles remained for cognitive dysfunction and 11 for major depressive disorder, which are presented and discussed in this study. Further research into non-motor symptoms of PD may provide insights into mechanisms of neurodegeneration, and provide better quality of life by using rational drugs.

## Introduction

Parkinson disease (PD) is one of the most complex neurodegenerative diseases with a broad spectrum of motor and non-motor symptoms (1). According to the United Kingdom Parkinson Disease Society Brain Bank, the clinical diagnosis is based on the presence of two or three motor features: bradykinesia plus rigidity or tremor at rest (or both). However, these criteria neither separate PD from the many other forms of parkinsonism nor contemplates the non-motor signs. Despite intense research, no effective therapy is currently available to prevent the onset, or to halt the progression of the disease.

In PD patients, there is a spectrum of cognitive dysfunction, ranging from mild cognitive impairment (PD-MCI) to PD dementia [PDD; (2)]. PD-MCI may represent the earliest stage of cognitive decline and a risk factor for developing PDD (3). Even when it is considered to be common, frequency estimates may vary between studies due to many factors such as differences in the populations studied (e.g., clinic or community-based; incident or prevalent of PD), clinical and neuropsychological criteria used, and the number and type of neuropsychological tests assessed (4). A comprehensive review of the literature on PD and cognitive impairment conducted by the movement disorder society (MDS) task force on PD-MCI demonstrated a mean cross-sectional prevalence rate of 26.7% (range 18.9–38.2%) in non-demented patients (5) and its association with the subsequent development of PDD (3). The clinical profile of PD-MCI is heterogeneous, with a broad spectrum of clinical deficits and severity affecting both non-amnestic and amnestic domains, such as executive function, psychomotor speed, visuospatial abilities, language, and memory. Overall, non-amnestic single-domain impairment is the most affected (4).

Previous studies indicated that executive deficits were more important predictors of subsequent cognitive decline (6, 7). More recently, authors of The CamPaIGN study reported a 10-year follow up in patients with PD; they found that a poorer baseline performance on tests with a posterior cortical basis resulted in an increased risk for dementia, which leaded to the distinction of two cognitive syndromes in PD: a “frontal executive” cognitive deficit, primarily due to dysfunction in dopaminergic frontostriatal networks; and a “posterior cortical” cognitive deficit due to dysfunction in non-dopaminergic systems, which were related to the development of dementia and characterized by deficits on semantic fluency and pentagon copying (8). One study supported by functional imaging, which have reported that patients with PDD presented with a severe cortical cholinergic deficit in comparison to non-demented PD patients, indicates that the acetylcholine system is the non-dopaminergic system related to the posterior cortical subtype and to the progression to dementia (9).

This diverse profile in MCI-PD (evident even in early phases) characterizes the clinical heterogeneity of cognitive impairment and its risk for subsequent dementia, being the most affected, the executive, mnemonic, and visuospatial domains (6, 10, 11).

Another systematic review showed that the prevalence of dementia among PD subjects, which included a total number of 1767 PD patients, 554 of them with dementia, was of 24.5% (95% confidence interval 17.4–31.5%; (12). The clinical profile of PDD includes impairment of the attention, memory, language, visuospatial function, construction, praxis, and executive function domains. There are some phenomenological differences between PDD and Alzheimer dementia (AD), particularly in executive functions, so that a “subcortical” or “dysexecutive” pattern predominates in PDD. However, these differences are difficult to identify in the late stages of dementia (13). Neuropathological studies, therefore, revealed that cortical Lewy body/neuritic pathology is more extensive and severe in PDD than in PD without dementia (14). Also, cholinergic deficits occur in PDD, with higher levels in those with a longer duration of parkinsonism prior to dementia with lower cortical and limbic Lewy body/neuritic burden (15), and are ascribed to neuronal loss in basal forebrain cholinergic nuclei (15, 16). The presence of cortical cholinergic deficit in patients with PDD suggests that treatment with cholinesterase inhibitors may be beneficial (17).

Given the chronic and debilitating nature of PD, it is not surprising that many patients suffer negative emotional consequences, particularly depression. Other psychiatric symptoms in PD include sleep disorders, cognitive impairment, psychosis, and anxiety (18). Reported rates of major depressive disorder in PD patients vary widely, ranging from 7 to 76% (18). Few scales like the Beck depression inventory (BDI) and the Geriatric Depression Scale of Yesavage (GDS) have been validated for the screening of depression in PD patients, or to assess the severity of it, which is the case of the Hamilton rating scale for depression (HAMD) and the Montgomery–Asberg depression rating scale [MADRS; (19)]. The etiology of depression in PD is thought to be an interaction of exogenous causes (e.g., the fact of being diagnosed with a disabling, chronic disease for which there is no known cure) and endogenous causes (e.g., dopamine deficiency) (20). As the quality of life (QoL) in PD patients can be affected by psychiatric symptoms, treatment for major depressive disorder in PD is clinically relevant (21).

Cognitive dysfunction and major depressive disorder are both recognized entities that can appear simultaneously in any step of neurodegeneration in PD. Up to day, there are no recommendations for the optimal treatment for any of these comorbidities of PD. Here, we evaluate available evidence from clinical studies to identify the efficacy and safety of available pharmacological options to treat cognitive dysfunction and major depressive disorder in PD.

## Methods

We followed the PRISMA model (Preferred Reporting Items for Systematic reviews and Meta-Analyses).

### Eligibility criteria

(a) *Studies*: to evaluate the efficacy and safety of pharmacological treatments on cognitive dysfunction and major depressive disorder in PD, we selected clinical trials, cross-sectional observational studies and case–control studies. However, only English language articles published from 2004 to 2014 were included.
(b) *Participants*: PD patients (>18 years old; both genders) with diagnostic of cognitive dysfunction or major depressive disorder according to the DSM-IV (diagnostic and statistical manual of mental disorders) criteria including depressed mood or a loss of interest or pleasure in daily activities for more than 2 weeks (mood represents a change from the person’s baseline); impaired function (social, occupational, educational); specific symptoms [at least five of the following nine present nearly every day: (1) depressed mood or irritability most of the day, as indicated by either subjective report or observation made by others; (2) decreased interest or pleasure in most activities, most of each day; (3) significant weight change (5%) or change in appetite; (4) change in sleep, whether insomnia or hypersomnia; (5) change in activity; (6) fatigue or loss of energy; (7) guilt/worthlessness; (8) concentration impairment: diminished ability to think or concentrate, or more indecisiveness; (9) suicidality: thoughts of death or having a suicide plan].
(c) *Intervention*: pharmacological therapy.
(d) *Outcome measures*: efficacy, safety, and QoL.

### Sources of information

We included articles from PubMed electronic database updated until May, 2014. MeSH terms (Medical subject headings) used for the search were “*parkinson disease*,” “*Delirium*,” “*Dementia*,” “*Amnestic*,” “*Cognitive disorders*,” “*depression*,” “*major depressive disorder*,” and “*drug therapy*.”

### Article selection

Articles were evaluated by filtering the studies through analysis of the title, followed by summary and critical analysis of the full article. To evaluate the methodological quality of the randomized clinical trials, we used the CONSORT criteria (Consolidated Standards of Reporting Trials) available on http://www.consort-statement.org.

### Data search

The following data were extracted from the selected articles: authors, type of study, participant’s characteristics, pharmacological intervention, diagnostic criteria, scales, efficacy of intervention, safety of the intervention, and QoL.

### Exclusion criteria

Studies without pharmacological treatment strategies were excluded. The selection and evaluation of the articles were made by two separated blinded authors.

### Bias risk assessment

We analyzed eligibility criteria for participants of the sample; the random allocation of participants, the presence of a control group, *the results from the analysis of more than 85% of the sample*, the presentation of results, and the inter group variability of the results. A sensitivity analysis of included data was not performed.

## Results

**Figure 1:**
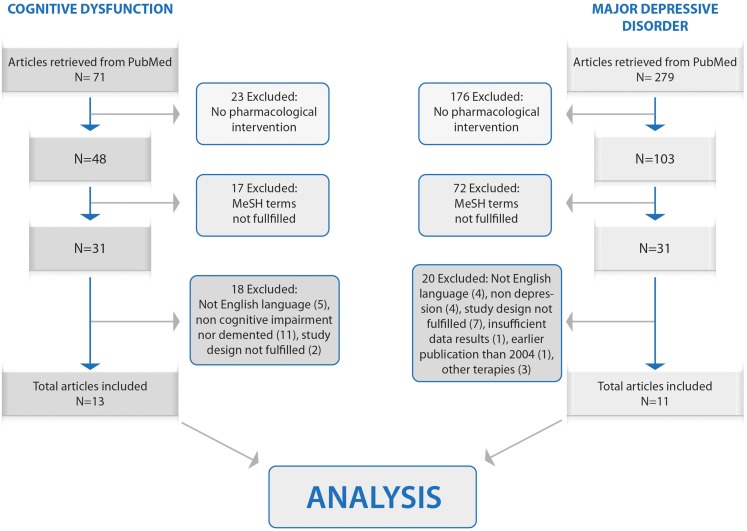
**Flowchart for articles selection**.

The search generated a total of 350 studies: 279 related to major depressive disorder and 71 to cognitive dysfunction. One hundred ninety-nine articles were excluded because of non-focus on pharmacological intervention; 89 were excluded because of non-satisfied MeSH selection criteria; 38 were excluded due to a poor/non-rigorous study design, publication date, use of other therapies and/or insufficient data. After all the inclusion and exclusion criteria were fulfilled, 24 articles remained: 13 for cognitive dysfunction and 11 for major depressive disorder. Those articles are presented and discussed in this study. The CONSORT flow diagram is shown in Figure 1.

### Cognitive dysfunction and Parkinson’s disease

For cognitive dysfunction in PD, 13 articles were analyzed (22–34). All of them were clinical trials (see Table 1). Neither cross-sectional observational studies nor case–control studies were found. All, except one, included patients with PDD. In total, 3853 patients were studied (see Table 1). All articles were obtained in full-text version and critically analyzed.

**Table 1:** **Rational pharmacological approaches for cognitive dysfunction in Parkinson’s disease**.

Reference	Study	Intervention	Population	Scales used	Efficacy	Safety	Quality of life
Emre et al. (22)	Clinical trial	Rivastigmine 3–12 mg/day	541 PDD patients	ADAS-cog, ADCS-CGIC, ADCS-ADL, NPI-10, MMSE, CDR, D-KEFS	Moderate significant efficacy in global ratings of dementia and cognition (*p* < 0.0005)	AE in 83.7% of patients in rivastigmine group. Mild to moderate events	Not evaluated
Poewe et al. (26)	Clinical trial	Rivastigmine 1.5–6 mg/day	334 PDD patients	ADAS-cog, ADCS-ADL, NPI-10, MMSE, D-KEFS	Improved cognitive performance above baseline for up to 48 weeks	AE in 75.4% of patients	Not evaluated
Burn et al. (25, 35)	Clinical trial	Rivastigmine 3–12 mg/day	536 PDD patients	ADAS-cog, ADCS-CGIC, ADCS-ADL, D-KEFS, CDR, MMSE, NPI-10	Greater benefits in patients with visual hallucinations (*p* < 0.005)	Nausea and vomiting were the most commonly AE	Not evaluated
Barone et al. (27)	Clinical trial	Rivastigmine 7.8–8.4 mg/day	342 PDD patients	ADAS-cog, ADCS-CGIC, ADCS-ADL, CDR	Patients with elevated homocysteine levels had greater effects on cognition (*p* < 0.01)	Severe AE were more commonly in those with elevated homocysteine	Not evaluated
Wesnes et al. (24)	Clinical trial	Rivastigmine 3–12 mg/day	541 PDD patients	CDR	Improvement on power of attention, continuity of attention, cognitive reaction time and reaction time (*p* < 0.01)	Not evaluated	Not evaluated
Schmitt et al. (32)	Clinical trial	Rivastigmine 3–12 mg/day	402 PDD patients	D-KEFS	Significant effects on tests of letter fluency, card sorting, and symbol digit modalities (*p* < 0.05)	Not evaluated	Not evaluated
Olin et al. (31)	Clinical trial	Rivastigmine 3–12 mg/day	402 PDD patients	ADCS-ADL	Modest beneficial effects on basic and higher level ADL functioning	Not evaluated	Not evaluated
Ravina et al. (23)	Clinical trial	Donepezil 5–10 mg/day	22 PDD patients	ADAS-cog, MMSE, DRS	Modest effect on cognitive function according to MMSE	AE in 52% of patients	Not evaluated
Dubois et al. (34)	Clinical trial	Donepezil 5–10 mg/day	550 PDD patients	ADAS-cog, CIBIC+	Evidence suggesting cognition and execution function improvement in Parkinson’s disease dementia	Higher incidence of adverse effects in the donepezil treated groups	Not evaluated
Litvinenko et al. (28)	Clinical trial	Galantamine 8–16 mg	41 PDD patients	MMSE, ADAS-cog, FAB, CDT, NPI-12, DAD	Positive effects on the overall level of cognitive impairments assessed on the MMSE and ADAS-cog scales (*p* < 0.05)	AE in 30% of patients	Not evaluated
Litvinenko et al. (30)	Clinical trial	Memantine 20 mg/day	62 PDD patients	MMSE, ADAS-cog, FAB, D-KEFS, CDR, NPI-12, DAD	Positive effects on the overall level of cognitive impairments assessed on the MMSE and ADAS-cog scales (*p* < 0.05)	AE in 3/32 patients	Not evaluated
Leroi et al. (29)	Clinical trial	Memantine 20 mg/day	25 PDD patients	DRS, NPI, MMSE, CIBIC-Plus	Statically significant benefit on MMSE	1 AE, unlikely to be related to the study medication	Not evaluated
Hanagasi et al. (33)	Clinical trial	Rasagiline 1 mg/day	55 PD-MCI patients	CDR, Stroop test, TMT A and B	Beneficial effects on certain aspects of cognition in tests of attention and executive functions (*p* < 0.05)	3 AE reported	Not evaluated

*Parkinson’s disease dementia (PDD), mild cognitive impairment (MCI), adverse events (AE), cognitive subscale of the Alzheimer’s disease assessment scale (ADAS-cog), Alzheimer’s disease cooperative study – clinician’s global impression of change (ADCS-CGIC), Alzheimer’s disease cooperative study activities of daily living (ADCS-ADL), 10-item neuropsychiatric inventory (NPI-10), mini-mental state examination (MMSE), computerized assessment system power of attention tests (CDR), verbal fluency test from the Delis–Kaplan executive function system (D-KEFS), clinician’s interview-based impression of change plus caregiver input (CIBIC+; global function), frontal lobe dysfunction assessment battery (FAB), neuropsychiatric inventory (NPI-12), disability assessment dementia (DAD), dementia rating scale (DRS), trail making test (TMT)*.

Pharmacological treatments used in these clinical studies were rivastigmine (3–12 mg) during 24 weeks, rivastigmine (1.5–6 mg) during 24 weeks, donepezil (5–10 mg) during 20 weeks, donepezil (5–10 mg) during 24 weeks, galantamine (8–16) during 24 weeks, memantine (20 mg) during 52 weeks, memantine (20 mg) during 22 weeks, and rasagiline (1 mg) during 12 weeks.

Cognitive dysfunction was defined as impairment in two out of four cognitive domains in screening neuropsychological tests. All but two studies used the DSM-IV diagnostic criteria for PDD (the other two used ICD-10). Seven of eight studies used structured interviews as mini international neuropsychiatric interview (MINI) or structured clinical interview (SCI). The main outcomes were efficacy, safety, and QoL.

To identify the efficacy, all clinical studies evaluated pharmacological treatment response and improvement of cognitive dysfunction; nevertheless, the authors used different scales: cognitive subscale of Alzheimer’s disease assessment scale (ADAS-cog), Alzheimer’s disease cooperative study – clinician’s global impression of change (ADCS-CGIC), Alzheimer’s disease cooperative study activities of daily living (ADCS-ADL), 10-item neuropsychiatric inventory (NPI-10), mini-mental state examination (MMSE), computerized assessment system power of attention tests (CDR), verbal fluency test from the Delis–Kaplan executive function system (D-KEFS), clinician’s interview-based impression of change plus caregiver input (CIBIC+; global function), frontal lobe dysfunction assessment battery (FAB), neuropsychiatric inventory (NPI-12), disability assessment dementia (DAD), dementia rating scale (DRS), trail making test (TMT), and nurses observation scale for geriatric patients (NOS).

### Major depressive disorder and Parkinson’s disease

Eleven articles were analyzed (20, 36–45). All of them were clinical trials (see Table 2). Neither cross-sectional observational studies nor case–control studies were found. In total, 510 patients were studied (Table 2). All articles were obtained in full-text version and critically analyzed.

Pharmacological treatments used in the studies were duloxetine (60 mg/day) during 12 weeks, nortriptyline (25–75 mg) during 8 weeks, paroxetine (continuous release 12.5–37.5 mg) during 12 weeks, citalopram (10–30 mg/day) during 12 weeks, Omega-3 (4 capsules/day) during 3 months, escitalopram (20 mg/day) during 12 weeks, pramipexole (1.5–4.5 mg/day) during 14 weeks, sertraline (50 mg/day) during 14 weeks, reboxetine (3.75–4.2 mg/day) during 4 months, rasagiline (1–2 mg/day) during 8 weeks, desipramine (75 mg/day), and citalopram (20 mg/day) during 14 and 30 days.

All the studies used the DSM-IV major depressive disorder diagnostic criteria for enrollment patients; 8 of the 11 studies also used structured interviews as MINI or SCI. The main outcomes to be measured were efficacy, safety, and QoL.

To identify the efficacy, those clinical studies evaluated the response to pharmacological treatments and remission of depression; nevertheless, the authors used different scales: HAMD (clinical response defined as a 50% reduction in baseline to endpoint score and remission defined as a <8 score), BDI, clinical global impression-severity (CGI-S), clinical global impression improvement scale (CGI-I), MADRS, inventory of depressive symptomatology (IDS), GDS, and the hospital anxiety and depression scale (HADS). One study evaluated efficacy through brain structural changes in single-photon emission computed tomography (SPECT).

Seven articles evaluated the safety of pharmacological therapy. Side effects were used as outcome measure. To assess the QoL some studies used the Nottingham quality of life scale (NHP), Parkinson disease questionnaire (PDQ8), and the short form health survey (SF-36).

Two clinical trials with PD patients and major depressive disorder evaluated the use of selective serotonin reuptake inhibitor (SSRIs) citalopram at doses of 10–30 mg/day. The treatment response was measured by cerebral blood flow using SPECT scan. Larger cortical areas were found to be involved in depressed PD patients with hyperactivity (reciprocal to basal degeneration in PD and maybe dopaminergic treatment) and with hypoactivity (probably due to organic lesions leading to hypoperfusion). Measurement of monoamines levels, brain-derived neurotrophic factor (BDNF), orexin-A, interleukin 6 (IL-6), and corticosterone in the cerebrospinal fluid reported low levels of BDNF and IL-6. Both studies registered improvement in depressive symptoms without reporting efficacy, side effects, and QoL. The treatment response using escitalopram (dose of 20 mg/day) was 37%; depression remission was 50%; 14% of the population reported side effects (nausea and confusion), but this study did not report effects on QoL.

From these 11 studies, 63% reported security. Duloxetine was the drug that reported higher percentage of side effects (20.5%). Main side effects were diarrhea, tremor, nausea, vomiting, drowsiness, syncope, visual hallucinations, decreased libido, and psychotic symptoms.

One study compared a dopamine agonist (pramipexole) versus SSRI (sertraline). Patients included in the study showed no motor symptoms; PD patients with major depressive disorder without motor symptoms treated with pramipexole had a better treatment response and remission of the major depressive disorder in 60% of patients with <10% of side effects compared with sertraline; no difference was observed in QoL between the two drugs.

Another study tested fatty acids (Omega-3) in combination with antidepressants: sertraline, tricyclic antidepressants, and trazodone. It found an efficacy >40% compared with the placebo group.

Treatment with rasagiline (MAO-B) was associated with improvement in mood, especially at doses of 2 mg. Desipramine (tricyclic antidepressant) and citalopram (SSRIs) were compared showing an improvement in the acute treatment on day 14 with the tricyclic antidepressant and improvement with both antidepressants in the 30th, but side effects were reported (bradykinesia with citalopram, erectile dysfunction, and orthostatic hypotension with desimipramine).

The analyzed studies, however, did not specify the major depressive disorder domains in which the drugs had an effect.

## Discussion

### Cognitive dysfunction

All except one trial included patients with PDD, situation which reflects the difficulties in designing studies in groups with heterogeneous cognitive profiles, such as those seen in MCI-PD and early stages of PDD.

Although rivastigmine appears to be the most consistent treatment, all clinical trials are from the same population studied by the same group (22). Even when the study of the same population allows to compare every clinical trial between each other and to get more confident conclusions about doses and duration of treatment, those may not be valid to other populations. Taking that into account, it may be recommendable to conduct clinical trials in different populations to compare results and to ensure a correct generalization of the conclusions.

**Table 2:** **Rational pharmacological approaches for major depressive disorder in Parkinson’s disease**.

Reference	Study	Intervention	Population	Scales used	Efficacy	Safety	Quality of life
Bonuccelli et al. (44)	Clinical trial	Duloxetine 60 mg	151 Patients	HAMD-17, BDI, CGI-S, PDQ-39	Response (60.4%) and remission (45.6%)	Adverse events reported in 20.5% of patients	Significant improvement (*p* < 0.001) on emotional well being, stigma, cognitive impairment, and bodily discomfort, as measured by PDQ-39
Dobkin et al. (43)	Clinical trial	Nortriptyline (25–75 mg), paroxetine (12.5–37.5 mg)	52 Patients: paroxetine 18, nortriptyline 17, placebo 17	HAMD-17, CGI-I, HAMA	Response to acute treatment in 16 patients: paroxetine (3), nortriptyline (9), placebo (4)	Not evaluated	Not evaluated
Palhagen et al. (42)	Clinical trial	Citalopram 10–30 mg/day	37 Patients: PD + MD (11), PD (14), MD (12)	HAMD-17, MADRS	Modest effect on cognitive function according to MMSE	Not evaluated	Not evaluated
Palhagen et al. (40)	Clinical trial	Citalopram 10–30 mg/day	37 Patients: PD + MD (11), PD (14), MD (12)	HAMD-17, MADR, SPECT	Expected decrease in the 5 HIAA and MHPG levels in patients with solely MD, but not in PD patients with MD. Levels of BDNF and IL-6 were lower in the PD patients	Not evaluated	Not evaluated
Da Silva et al. (20)	Clinical trial	Omega-3 4 capsules	17 Patients: Omega-3 (7), placebo (10)	MADRS, BDI, CGI	Positive effects on the overall level of cognitive impairments assessed on the MMSE and ADAS-cog scales (*p* < 0.05)	Not evaluated	Not evaluated
Weintraub et al. (38)	Clinical trial	Escitalopram 20 mg	14 Patients	HAMD, IS, CGI-I	42% of patients responded	Adverse events reported in 2 patients (nausea and confusion)	Not evaluated
Barone et al. (36)	Clinical trial	Pramipexole 1.5–4.5 mg/day, sertraline 50 mg/day	67 Patients	HAMD-17, SF-36	Larger cortical areas were found to be involved in depressed PD patients, both with hyperactivity and with hypoactivity	Adverse events reported in 3 patients on the pramipexole group and in 8 on the sertraline group	General improvement for both groups
Pintor et al. (37)	Clinical trial	Reboxetine 3.74–4.2 mg/day	17 Patients	HAMD, GDS, HADS, NHP	Improvement of 50% at the HAMD scores in 12 patients. HAD mean scores decreased by 59.34 and 52.01% at the GDS.	Adverse events reported in 2 patients (vertigo and redness)	Mean scores on the NHP decreased by 44.59%
Korchounov et al. (45)	Clinical trial	Rasagiline 1–2 mg/day	6 Patients	HAMD-17	Subjects treated with 2 mg/d scored <14 after treatment at HDRS score	Adverse effects or insomnia were not reported	Improvement was more pronounced if treated with 2 mg of rasagiline (*p* = 0.20)
Reference	Study	Intervention	Population	Scales used	Efficacy	Safety	Quality of life
Devos et al. (39)	Clinical trial	Placebo 3 tablets Citalopram 20 mg/day Desipramine 75 mg/day	16 Patients 15 Patients 17 Patients	MADRS	After 14 days, desipramine prompted an improvement in the MADRS score, compared with citalopram and placebo. Both antidepressants produced significant improvements after 30 days	Mild adverse events: bradykinesia, erectile dysfunction and worsened orthostatic hypotension	Not evaluated
Menza et al. (41)	Clinical trial	Nortriptyline (25–75 mg), paroxetine (12.5–37.5 mg)	52 Patients: paroxetine (18), nortriptyline (17), placebo (17)	HAMD-17, CGI-I, HAMA, SCI, SF-36, PDQ8	30 Patients showed improvement	Generally mild or moderate	Improvement when patients demonstrated improvement in depression

*Parkinson disease (PD), mood disorder (MD), Omega-3 (W3), antidepressant (AD), Hamilton rating scale for depression (HAMD), Hamilton anxiety rating scale (HAMA), mini international neuropsychiatric interview (MINI), Beck depression inventory (BDI), clinical global impression-severity (CGI-S), structured clinical interview (SCI), clinical global impression improvement scale (CGI-I), Montgomery–Asberg depression rating scale (MADRS), single-photon emission computed tomography (SPECT), inventory of depressive symptomatology (IDS), Geriatric Depression Scale of Yesavage (GDS), hospital anxiety and depression scale (HADS), the short form health survey (SF-36), Parkinson disease questionnaire (PDQ8), and quality of life scale: Nottingham (NHP)*.

It is important to note that even though the multinational trial by Dubois et al. with donepezil did not meet its planned primary objective, the alternative ADAS-cog analysis while removing the treatment-by-country interaction from the model, revealed a significant, dose-dependent benefit with donepezil. Authors attributed this to possible imbalance in enrollment between participant countries (34). This finding is consistent with clinical practice, where caregivers report improvement in cognition of their patients when donepezil is prescribed.

Patients with PDD were included according to the DSM-IV criteria (which requires the presence of memory impairment), and while analyzing the efficacy of treatment, we found that most primary outcomes were assessed with ADAS-cog (see Table 1), a scale designed to assess the severity of the major symptoms of Alzheimer. The main areas of the cognitive domains evaluated with this scale are memory (50%), language (28%), praxis (14%), and command understanding (8%) (46); however, this scale lacks specificity for PDD due to obvious differences in the profiles of these two conditions; the predominance of memory impairment in the first one and the “dysexecutive” impairment on the second one. The fact that patients were included with the DSM-IV criteria and that the instrument utilized to assess the primary end point of efficacy was the ADAS-cog makes that the results of the studies focused mainly in domains usually affected in Alzheimer’s disease but not in those related with the cognitive profile in PDD.

Comparative studies used in the task force for clinical diagnostic criteria for dementia associated with Parkinson’s disease matched dementia on the basis of the DRS. Therefore, it has been suggested as an alternative to assess efficacy in clinical trials with cholinesterase inhibitors for PDD, given its sensitivity for the diagnosis of executive dysfunction (47). This scale was used as a part of the secondary objective in two studies of the current review.

Task force has made recommendations about the test for use in the diagnosis of PDD (48) and PD-MCI (2). Measures specific to PD have the advantage that they emphasize testing cognitive deficits associated with PD (e.g., executive and visuospatial deficits) whereas generic measures tend to focus on memory abilities. Scales designed to asses cognitive impairment associated with PD include the Parkinson’s disease dementia – short screen (PDD-SS) (49), the Parkinson neuropsychiatric dementia assessment (PANDA) (50), the mini-mental Parkinson (MMP) (51), the scales for outcomes of Parkinson’s disease – cognition (SCOPA-Cog) (52), and the Parkinson’s disease cognitive rating scale (PD-CRS) (53). All of them with good diagnostic accuracy and some need a minimum of time for administration. Formal assessment of each domain requires more detailed neuropsychological tests. Future studies will benefit from using these ones and will allow more definitive conclusions.

When conducting clinical trials about treatment for cognitive dysfunction, it is important to take into account the presence of neuropsychiatric symptoms; depression, apathy, anxiety, and irritability, are frequent in non-demented PD patients (54). The first two can affect the cognitive performance on neuropsychological tests since they have been negatively associated with executive functioning and with immediate memory (55). Although not all, most of the studies of the current review, state that the presence of depression was an exclusion criterion, partly eliminating in this way potential sources of confusion.

The association between cognitive dysfunction and depressive symptoms has a possible underlying mechanism in common, which in some studies has been related to acetylcholine. In this respect, Bohnen et al. demonstrated a significant inverse correlation between cortical AChE activity and the scores of the Cornell scale for depression in dementia. This correlation remained significant after controlling for mini-mental state examination scores (56). Moreover, Meyer et al. reported *in vivo* reductions of α4β2 nAChRs in PD that correlated with both increased severity of depressive symptoms and severity of cognitive symptoms (57).

Studies indicate that the clinical phenotype is also associated with dementia, patients with a tremor-dominant phenotype are rare to present dementia (58), while in patients with axial symptoms dementia is developed earlier in the course of the disease (35). Moreover, in a recent meta-analysis, patients with non-tremor predominant motor symptoms had more severe cognitive impairments than tremor-dominant patients. Results of this study also suggested that PD subgroups with depression had more severe cognitive impairment. Because of the influence of depression and the subtype of predominant motor symptoms on cognition authors consider important to take them into account when evaluating a cognitive profile in PD (59). It would be interesting to determine if certain clinical phenotype benefits more from therapy, which also could indicate or reinforce the implicated mechanisms of cognitive dysfunction in these patients.

Nevertheless, none of the studies assessed QoL (see Table 1). QoL is nowadays considered a unique and irreplaceable assessment for clinical evaluation. Instead of QoL, the ADCS-ADL scale was used. This scale can indirectly quantify the QoL of patients and care givers. However, the activities assessed by the scale can be related to both, cognitive impairment and motor symptoms, which can be a confounder if appropriate measures are not taken.

Although the unified Parkinson’s disease rating scale (UPDRS) was assessed before and after each study, the ON/OFF status of the patient is not mentioned during the neuropsychological assessment of the patient, which can influence the scores that require motor responses.

Unfortunately, only one study included patients with PD-MCI, it revealed that rasagiline may confer some beneficial effects on certain aspects of cognition in this patient population (33). The relevance of the PD-MCI as a risk factor for the development of PDD, makes this study a start point for future studies aiming to treat and prevent PDD. New clinical criteria for diagnosing PD-MCI will allow uniform inclusion for PD patients’ in new clinical trials.

In spite of this limitations, studies showed benefit in the primary endpoint for rivastigmine and if removing the treatment-by-country interaction, also for donepezil. A recent study assessing the efficacy of cholinestarease inhibitors in PDD, MCI-PD, and Lewy body dementia concluded that the beneficial effect on cognitive function was observed in both the donepezil and rivastigmine groups (SMD −0.42, 95% CI −0.58 to −0.25, *p* < 0.00001; SMD −0.27, 95% CI −0.44 to −0.11, *p* < 0.001, respectively) (60).

In the near future, it would be interesting to conduct functional studies in combination with biomarkers in order to determine additional mechanisms for PDD.

### Major depressive disorder

All articles reviewed in this study aimed to treat major depressive disorder in PD patients mainly by the use of SSRIs antidepressants. This type of antidepressants is often used in clinical practice, despite possible side effects as nausea, vomiting, sexual dysfunction, diarrhea, and auditory hallucinations (21).

Two clinical trials included in this review reported response to citalopram treatment measured by cerebral blood flow by SPECT (40) and monoamine levels, BDNF, orexin-A, IL-6, and corticosterone in the cerebrospinal fluid (42), but did not study side effects. These studies only reported an improvement in depressive symptoms without outcome measures as efficacy, side effects or QoL (see Table 2). One study reported side effects with citalopram (bradykinesia) (39).

A study treating patients with dopamine agonists reported a reduction of depression symptoms; however, the mechanism of this reduction is not clear (36). We could suggest an indirect effect associated with improvement on motor symptoms. The authors compared pramipexole with sertraline to treat major depressive disorder in PD patients without history of motor symptoms. The pramipexole group had a greater response to treatment, remission of depression symptoms, and fewer side effects compared with the sertraline group (36).

The antidepressant effect of rasagiline was obtained with higher doses than those used for motor control symptoms (1 mg/day). The authors suggesting that the improvement in mood is not a direct result of the improvement in motor function.

Selective serotonin reuptake inhibitor, serotonin–norepinephrine reuptake inhibitors (SNRIs), selective noradrenaline recapture inhibitor, and tricyclic antidepressants (TCAs) seem to be a good strategy to treat PD patients. Nevertheless, the non-homogeneous methodology used in the studies to assess outcome measures and the limited number of studies focused on these drugs complicate a statistical analysis and getting a conclusion by a meta-analysis. SSRIs are the most studied drugs, reporting a high rate of response to treatment and remission of the symptoms associated with major depressive disorder (21). The American Academy of Neurology declared that there is insufficient evidence to recommend specific antidepressant treatments for major depression disorder in PD patients (61). Considering that this is a chronic and severe disability condition, the economic impact for the patient suffering from this condition is high. Therefore, the development of a treatment guideline for patients with PD and major depressive disorder is necessary.

## Conclusion

Here, we ask the question “are these current pharmacological strategies safe and efficient to treat cognitive dysfunction and depression in Parkinson’s disease?”

Unfortunately not! There is insufficient evidence to recommend specific pharmacological treatment for these non-motor symptoms of PD. Therefore, there is the need to conduct more clinical studies.

Finally, we must remember that, however, exciting the neurobiological mechanisms might be, the clinical usefulness of rational therapeutic approaches will be determined by their ability to provide efficacy, safety, long-lasting, and substantial improvements in QoL.

Most of the studies of cognitive dysfunction in Parkinson have been conducted in patients with dementia; however, the different prognosis in syndromes of cognitive impairment makes mandatory to develop studies since these stages where clinical trials are lacking.

Early MCI and PDD differences in cognitive profile will allow that disease modifying therapies may be targeted at the different dopaminergic and non-dopaminergic mechanisms involved in the pathophysiology of cognitive dysfunction in patients with Parkinson’s disease.

It is necessary to design clinical trials according to criteria and end point measures specifically designed for MCI-PD and PDD, which will allow more definitive conclusions and understanding in this topic.

## Conflict of Interest Statement

The authors declare that the research was conducted in the absence of any commercial or financial relationships that could be construed as a potential conflict of interest.
